# The safety and efficacy of Ezetimibe Plus Statins on ASVD and Related Diseases

**DOI:** 10.14336/AD.2021.0412

**Published:** 2021-12-01

**Authors:** Shuling Wan, Yuchuan Ding, Xunming Ji, Ran Meng

**Affiliations:** ^1^Department of Neurology, Xuanwu Hospital, Capital Medical University, Beijing, China.; ^2^Advanced Center of Stroke, Beijing Institute for Brain Disorders, Beijing, China.; ^3^Department of China-America Institute of Neuroscience, Xuanwu Hospital, Capital Medical University, Beijing, China.; ^4^Department of Neurosurgery, Wayne State University School of Medicine, Detroit, Michigan, USA.

**Keywords:** ezetimibe, statin, atherosclerotic vascular disease

## Abstract

It is well known that atherosclerotic vascular disease (ASVD) in the elderly is a global disease with high morbidity, mortality and disability, and plasma LDL-C correction is the most important strategy for ASVD control. However, a large proportion of patients failed to achieve their ideal LDL-C goals after statins use. Ezetimibe, a newly non-statin lipid-lowering agent, is an inhibitor of exogenous cholesterol absorption. Whereby, ezetimibe plus statins may reduce LDL-C more strongly than statins alone. Differed from any other papers published previously, which only involved ezetimibe plus statins for coronary heart disease, the highlight of this paper is to summarize the efficacy and safety of ezetimibe plus statins in all kinds of ASVD subtypes and their related diseases, mainly included aortic atherosclerosis, coronary artery disease, cerebrovascular and peripheral artery diseases. Obviously, this paper is inimitable, which will provide the readers an important reference, especially in treating the elderly with multi-organs atherosclerosis.

Atherosclerotic vascular disease (ASVD) is very common in the elderly, included the subtypes of aortic atherosclerosis (AA), cerebrovascular disease (CVD), coronary heart disease (CHD) and peripheral artery disease (PAD), with high morbidity, mortality and disability worldwide [1]. Low-density lipoprotein cholesterol (LDL-C) plays a key role in the pathological process of ASVD. Decreasing plasma LDL-C to the target level is an important strategy for ASVD control. The National Cholesterol Education Program Adult Treatment Panel III (NCEP ATP III) advocated using the Framingham risk scoring system to estimate 10-year-risk for coronary event in patients. Each of the major CHD risk factors, including age, gender, total cholesterol, systolic blood pressure, high-density lipoprotein cholesterol (HDL-C), and smoking status, represented a certain score, and the sum yielded an estimated risk for suffering from a coronary event in 10 years. Patients with established CHD and CHD equivalents (diabetes, peripheral vascular disease, symptomatic carotid artery disease, and abdominal aortic aneurysm) were considered as in high-risk category (10-year CHD risk >20%), with two or more major risk factors were in moderate-risk category (10-year CHD risk 10% to 20%), and with one or fewer major risk factor were in low-risk category (10-year CHD risk <10%), and the goals of LDL-C control for patients in the three categories were <100 mg/dl, <130 mg/dl, and <160 mg/dl, respectively [2].

Statins, the inhibitors of 3-hydroxy-3-methylglutaryl-coenzyme-A (HMG-CoA) reductase (Fig. 1), are a class of agents most commonly used on lipid lowering and the cornerstone on primary and secondary prevention and treatment of both CVD and CHD [3-8]. Statins significantly inhibit endogenous cholesterol synthesis, reduce the level of plasma LDL-C, stabilize and regress plaques [9]. However, there are several limitations and concerns in statins use (Table 1). To begin with, some statin users encounter statin intolerance and discontinuation due to its adverse effects [10]. Generally, the higher the statin dose, the higher the incidence of adverse effects [11, 12]. Secondly, even though widespread use of statins, a great ratio of patients at high risk is unable to reach their LDL-C goals as NCEP ATP III recommended [13, 14]. For these patients, up titrating statin dosage or switching to a more potent statin may be beneficial. However, for each doubling of the statin dose, there is merely an additional 6-8% reduction in LDL-C [15, 16]. Thirdly, many statin-treated patients still possess a high ‘residual risk’ of cardiovascular (CV) events. The residual risk may partially be due to the insufficient LDL-C lowering, given that the risk reduction of CV events is proportional to the extent of the reduction in LDL-C [3, 4, 17-19]. Whereby, novel therapies or combination therapies are needed to further lower plasma LDL-C level, improve goal attainment and reduce ASVD.

**Figure 1.**
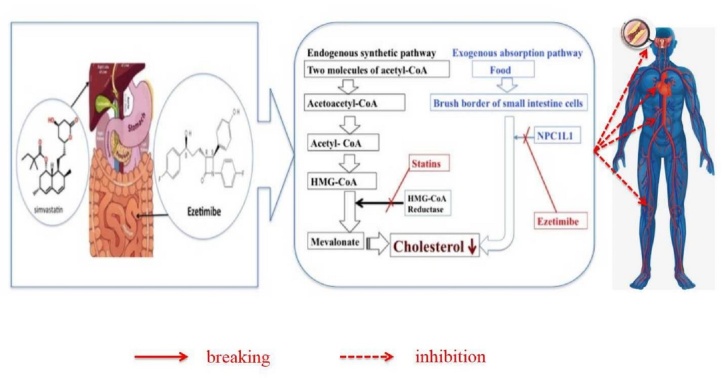
The schematic diagram of the targets of ezetimibe and statins.

Ezetimibe is a potent cholesterol absorption inhibitor targeting the Niemann-Pick C1-like 1 (NPC1L1) protein [20]. Ezetimibe prohibits the absorption of dietary and biliary cholesterol from the intestine but does not influence the uptake of triglycerides or fat-soluble vitamins (Fig. 1) [21]. The recommended dose of ezetimibe is 10 mg once daily, which can be orally administered in the morning or evening without regard to food. The pharmacokinetics of ezetimibe is not affected by age, sex or race and it is not necessary to adjust dosage in patients with mild hepatic impairment or mild-to-severe renal insufficiency. Most importantly, ezetimibe has a favorable drug-drug interaction profile, without significant effects on plasma levels of statins (atorvastatin, simvastatin, rosuvastatin, pitavastatin, lovastatin, fluvastatin, and pravastatin). Besides, there is no significant effect of concomitant administration of statins on ezetimibe bioavailability [22]. Whereby, adding ezetimibe to statins may reduce LDL-C remarkably, enhance goal attainment, decrease the dosage of statins use so as to decrease its adverse effects, and decrease the incidence of ASVD. This article aimed to evaluate the efficacy and safety of ezetimibe combined with statin therapy in patients with ASVD and related diseases.

## 1. The efficacy of ezetimibe plus statin therapy

### 1.1 The efficacy of ezetimibe plus statins on LDL-C lowering

Much evidence revealed that ezetimibe combined with statins could be more effectively to make plasma LDL-C levels to the goal than statins alone with both routine and high doses (Table 2) [23-40]. The Ildong rosuvastatin & ezetimibe for hypercholestelolemia study (I-ROSETTE; NCT02749994) compared the effects of fixed-dose combinations of rosuvastatin and ezetimibe (combination group) with rosuvastatin alone (monotherapy group) on LDL-C control in 396 Korean patients with hyper-cholesterolemia (approximately 83.5% had CHD or CHD risk equivalents) [39]. In this Phase III clinical trial, patients were randomized in a 1:1:1:1:1:1 ratio to receive rosuvastatin 5, 10, and 20 mg or corresponding dose rosuvastatin plus ezetimibe 10 mg. After 8-week treatment, the LDL-C reduction and goal attainment in the total combination group were much greater than that in the total monotherapy group (-57.0% vs. -44.4%, 92.3% vs. 79.9%, respectively; both P<0.001). In particular, compared to each corresponding dose of rosuvastatin alone, the combination therapy produced a greater reduction in LDL-C levels, with mean percent change more than 50%. In addition, the co-administration of ezetimibe with rosuvastatin 5 mg provided greater LDL-C lowering compared to rosuvastatin 20 mg (-51.6% vs. -49.2%), suggesting that ezetimibe plus a low-dose statin in patients with hypercholesterolemia can produce a comparable or greater reduction in LDL-C compared to a high-dose statin alone, which was consistent with the results from other randomized controlled trials [34-38]. Kim et al. showed that ezetimibe 10 mg plus rosuvastatin 5 mg resulted in larger, but not significant, LDL-C reduction than rosuvastatin 10 mg or 20 mg alone (-51.83% vs. -46.79%; P=0.465, -51.83% vs. -47.96%; P=0.572, respectively) [38]. Kerzner et al. confirmed that the co-administration of ezetimibe with each dose of lovastatin produced significantly (P<0.01) greater LDL-C reduction than the corresponding dose or the next higher dose of lovastatin alone, and the mean percentage of LDL-C decreasing induced by ezetimibe plus low dose of lovastatin (10 mg) was comparable to that induced by high dose (40 mg) of lovastatin alone (-33% vs. -29%; P=0.10) [36]. Melani et al. indicated that ezetimibe plus pravastatin 10 mg produced a larger degree of LDL-C reduction compared with the high dose (40 mg) of pravastatin alone (-34% vs. -29%; P≤0.05) [37]. Bays et al. revealed that the combination of ezetimibe and simvastatin provided a significant LDL-C reducing compared with the next higher dose of simvastatin alone (P<0.00l) [34]. Zieve and colleagues found that ezetimibe plus atorvastatin 10 mg more significantly decreased LDL-C at 6 and 12 weeks than doubling or quadrupling dose of atorvastatin alone in elder than 65-year patients with high risk for CHD [27].

**Table 1:** Pros and cons of statins versus statins combined with ezetimibe.

	Statins	Statins combined with ezetimibe
Pros	LDL-C reduction	Decrease LDL-C intensively
	Pleiotropic effects	Further reduce the incidence of ASVD
	Anti-inflammatory	Attenuate statin-related adverse effects
	Immunomodulatory	
	Anti-thrombosis	
	Vascular protection	
	Reduce the incidence of ASVD	
Cons	Statin intolerance and discontinuation	Cancer ^*^
	Myopathy	
	Liver toxicity	
	Increase the risk of diabetes	
	Cognitive impairment ^*^	
	Increase the risk of hemorrhagic stroke ^*^	

LDL-C, low-density lipoprotein cholesterol; ASVD, atherosclerotic vascular disease.

* uncertain

The Ezetimibe Add-On to Statin for Effectiveness (EASE) Trial is a community-based trial with a diverse population of patients [16]. The 6-week trial recruited 3,030 hypercholesterolemic patients whose LDL-C levels did not meet the NCEP ATP III targets, more than three-quarters of whom had CHD or CHD risk equivalents. Subjects were randomized to receive ezetimibe 10 mg or placebo to be added to their ongoing statin therapy (various types and a range of dosages). In total population, the addition of ezetimibe resulted in an additional 25.8% reduction in LDL-C, compared with an additional 2.7% reduction in placebo group (P<0.001); the percentage of LDL-C goal attainment is significantly higher in ezetimibe group than in placebo group (71.0% vs. 20.6%; P<0.001). More importantly, significant additional improvements in LDL-C lowering and goal attainment were seen in various subgroups (NCEP ATP III risk category, age, sex, race, statin brand and dose). The IN-CROSS study, a Phase IV randomized active-controlled 6-week study, assessed the efficacy of switching from ongoing statin monotherapy to ezetimibe 10 mg plus simvastatin 20 mg or rosuvastatin 10 mg alone in 618 hypercholesterolemic patients who at high risk for cardiovascular disease and failed to attain LDL-C goals [41]. Despite prior administration of statin monotherapy, ezetimibe plus simvastatin produced greater additional LDL-C reduction (27.7% vs. 16.9%; P≤0.001) and higher percentage of patients who reached LDL-C <100 mg/dl (72.5% vs. 56.2%), <77 mg/dl (38.0% vs. 18.9%) and <70 mg/dl (25.2% vs. 11.1%) than rosuvastatin alone. The 12-week, multicenter, randomized, double-blind, 5-arm parallel-group trial of Vytorin in the Elderly (VYTELD) evaluated the efficacy and safety of the usual starting dose of ezetimibe/simvastatin (10/20 mg) versus atorvastatin 10 or 20 mg and the next higher dose of ezetimibe/ simvastatin (10/40 mg) versus atorvastatin 40 mg in 1,289 hypercholesterolemic patients >65 years of age with moderately high/high risk for CHD [42]. Subjects randomized to ezetimibe/simvastatin had greater reduction in LDL-C than atorvastatin alone. The percentage of high-risk patients achieved LDL-C <70 mg/dl and percentage of moderate-risk patients achieved LDL-C <100 mg/dl in ezetimibe/simvastatin group were significantly higher than that in atorvastatin alone group, respectively. A large-scale retrospective observational study enrolled 27,919 statin-treated patients with CHD or CHD-risk equivalents, 9.6% of them switched to ezetimibe plus simvastatin therapy (10/10, 10/20, 10/40, 10/80 mg), 39.5% switched to uptitrated statin therapy (a higher dose of the same statin or a higher potency dose of another statin), and 50.9% continued with their initial statin therapy, the results demonstrated that patients who switched to ezetimibe plus simvastatin therapy were accompanied by greater LDL-C lowering and higher goal attainment, as compared with participants who received uptitrated statin therapy or initial statin therapy [43]. To sum up, the combination therapy of ezetimibe plus statins is better than statins alone on LDL-C control.

**Table 2:** Mean percent change in LDL-C and goal attainment rate in ezetimibe plus statins studies.

Study	Design	Treatment group (T)	Control group (C)	Mean baseline LDL (mg/dl)		Mean percent Change (%)			Goal Attainment (%)		
				T	C	T	C	P-value	T	C	P-value
**Ezetimibe plus statins versus the same dose of statins**											
Bays et al. 2004 [34]	RCT 12-week	EZE/SIMVA	SIMVA								
		Total (n=622)	Total (n=609)	176.2	177.5	-53.0	-39.0	<0.001	78.6	45.9	<0.001
		10/10 mg	10 mg			-44.8	-32.7	<0.001			
		10/20 mg	20 mg			-51.9	-34.2	<0.001			
		10/40 mg	40 mg			-55.2	-40.6	<0.001			
		10/80 mg	80 mg			-60.2	-48.5	<0.001			
Graaf et al. NCT00129402 2008 [35]	RCT 6-week	EZE/SIMVA	SIMVA								
		Total (n=126)	Total (n=122)	225.2	218.6	-49.5	-34.4	<0.01	NA	NA	NA
		10/10 mg	10 mg			-46.7	-30.4	<0.01			
		10/20 mg	20 mg			-49.5	-34.3	<0.01			
		10/40 mg	40 mg			-52.1	-38.6	<0.01			
Kerzner et al. 2003 [36]	RCT 12-week	EZE/LOVA	LOVA								
		Total (n=192)	Total (n=220)	176.0	178.0	-39.0	-25.0	<0.01	80.0	59.0	<0.01
		10/10 mg	10 mg			-33.0	-19.0	<0.01			
		10/20 mg	20 mg			-39.0	-26.0	<0.01			
		10/40 mg	40 mg			-45.0	-29.0	<0.01			
Melaniet al. 2003 [37]	RCT 12-week	EZE/PRAVA	PRAVA								
		Total (n=204)	Total (n=205)	178.0	178.0	-37.7	-24.3	<0.01	82.0	60.0	<0.01
		10/10 mg	10 mg			-34.0	-20.0	<0.01			
		10/20 mg	20 mg			-38.0	-24.0	<0.01			
		10/40 mg	40 mg			-41.0	-29.0	<0.01			
Kim et al. NCT03288038 2018 [38]	RCT 8-week	EZEROSUVA	ROSUVA								
		Total (n=188)	Total (n=187)	160.6	160.0	-56.5	-45.2	<0.01	94.2	86.6	0.0142
		10/5 mg (n=64)	5mg (n=62)	164.8	158.6	-51.8	-40.8	<0.01	95.3	79.0	0.0109
		10/10 mg (n=60)	10 mg (n=62)	157.3	160.6	-55.8	-46.8	<0.01	95.0	90.3	0.3307
		10/20 mg (n=64)	20 mg (n=63)	159.6	160.9	-61.8	-48.0	<0.01	92.2	90.5	0.6960
Hong et al. NCT02749994 2018 [39]	RCT 8-week	EZE/ROSUV	ROSUVA								
		Total (n=195)	Total (n=194)	153.5	151.6	-57.0	-44.4	<0.001	92.3	79.9	<0.001
		10/5 mg (n=65)	5 mg (n=65)	160.7	156.1	-51.8	-40.5	<0.001	86.2	67.7	
		10/10 mg (n=66)	10 mg (n=65)	146.5	146.0	-55.8	-44.4	<0.001	93.9	93.9	
		10/20 mg (n=64)	20 mg (n=64)	153.5	152.8	-62.2	-47.1	<0.001	96.9	82.8	
**Ezetimibe plus statins versus doubling the dose of statins**											
Averna et al. NCT00423579 2010 [23]	RCT 6-week	EZE/SIMVA 10/20 mg ( n=56)	SIMVA 40 mg (n=56)	125.9	128.0	-27.0	-12.0	<0.001	73.0	25.0	<0.001
Bardini et al. NCT00423488 2010 [24]	RCT 6-week	EZE/SIMVA 10/20 mg (n=37)	SIMVA 40 mg (n=50)	127.4	123.6	-32.2	-20.8	<0.01	78.4	60.0	0.052
Rosen et al. NCT00862251 2013 [25]	RCT 6-week	EZE/SIMVA 10/20mg (n=322)	SIMVA 40 mg or ATORVA 20 mg ^†^ (n=162)	98.9	97.3	-23.1	-8.4	<0.001	54.5	27.0	<0.001
			ROSUVA 10 mg (n=324)		97.4		-19.3	0.060		42.5	<0.001
Yu et al. NCT00652327 2012 [26]	RCT 8-week	EZE 10 mg + SIMVA 20mg or ATORVA 10 mg or PRAVA 20 mg (n=29)	SIMVA 40mg or ATORVA 20 mg or PRAVA 40 mg (n=34)	144.6	130.9	-26.2	-17.9	0.0026	58.6	41.2	0.1675
Zieve et al. NCT00418834 2010 [27]	RCT 6-week	EZE/ATORVA 10/10 mg (n=515)	ATORVA 20 mg (n=515)	103.0	101.0	-27.0	-13.0	<0.001	47.4 ^‡^	17.9 ^‡^	<0.001
Teramoto et al. NCT00871351 2012 [28]	RCT 12-week	EZE/ATORVA 10/10 mg (n=47)	ATORVA 20 mg (n=46)	145.3	146.8	-25.8	-15.1	<0.001	78.7	41.3	<0.001
			ROSUVA 2.5 mg (n=32)		144.6		0.8	<0.001		3.1	<0.001
Conard et al. NCT00276458 2008 [29]	RCT 6-week	EZE/ATORVA 10/20 mg (n=92)	ATORVA 40 mg (n=92)	120.0	118.0	-31.0	-11.0	<0.001	84.0	49.0	<0.001
Wu et al. 2018 [30]	RCT 12-week	EZE/ATORVA 10/20 mg (n=48)	ATORVA 40 mg (n=50)	128.1	123.8	NA	NA	NA	79.2 ^‡^	50.0 ^‡^	0.016
Hing Ling et al. NCT00782184 2012 [31]	RCT 6-week	EZE/SIMVA 10/40 mg (n=120)	ATORVA 40 mg ^†^ (n=130)	122.0	119.0	-26.8	-11.8	<0.001	69.2	41.3	<0.001
Leiter et al. NCT00276484 2008 [32]	RCT 6-week	EZE/ATORVA 10/40 mg (n=288)	ATORVA 80 mg (n=291)	89.0	90.0	-27.0	-11.0	<0.001	74.0	32.0	<0.001
Bays et al. NCT00783263 2011 [33]	RCT 6-week	EZE/ROSUVA	ROSUVA								
		Total (n=219)	Total (n=217)	NA	NA	-21.0	-5.7	<0.001	59.4	30.9	<0.001
		10/5 mg (n=98)	10 mg (n=96)	107.0	102.0	-17.9	-5.6	<0.001	55.1	31.3	<0.001
		10/10 mg (n=121)	20 mg (n=121)	101.0	98.0	-23.7	-6.3	<0.001	62.8	30.6	<0.001
**Other ezetimibe plus statins trials**											
Florentin et al. NCT00932620 2011 [40]	RCT 2-month	EZE/SIMVA 10/10 mg (n=50)	SIMVA 40 mg (n=50)	178.0	172.0	-49.0	-43.0	<0.05	NA	NA	NA
Farnier et al. NCT00479713 2009 [41]	RCT 6-week	EZE/SIMVA 10/20 mg (n=314)	ROSUVA 10 mg (n=304)	124.0	125.0	-27.7	-16.9	≤0.001	72.5	56.2	≤0.001
Foody et al. NCT00535405 2010 [42]	RCT 12-week	EZE/SIMVA 10/20 mg (n=232)	ATORVA 10 mg (n=242)	166.0	167.0	-54.2	-39.5	<0.001	83.6	58.7	<0.001
			ATORVA 20 mg (n=238)		165.0		-46.6	<0.001		76.9	<0.05
		EZE/SIMVA 10/40 mg (n=236)	ATORVA 40 mg (n=239)	163.0	168.0	-59.1	-50.8	<0.001	90.3	79.5	<0.05
Pearson et al. 2005 [16]	RCT 6-week	EZE 10 mg + Statin (n=2020)	Placebo + Statin (n=1010)	129.0	129.0	-25.8	-2.7	<0.001	71.0	20.6	<0.001
Toth et al. 2014 [43]	ROS	EZE/SIMVA (n=2671)	Statins titrated (n=11035)	110.4	104.7	-24.0	-9.6	<0.001	81.2	68.0	NA
			Statins not titrated (n=14213)		86.3		4.9	<0.001		72.2	NA
Tsujita et al. NCT01043380 2015 [45]	RCT 9 to 12-month	EZE 10 mg + ATORVA (n=122)	ATORVA (n=124)	109.8	108.3	-40.0	-29.0	<0.001	NA	NA	NA

RCT, randomized controlled trial; ROS, retrospective observational study; EZE, ezetimibe; SIMVA, simvastatin; ATORVA, atorvastatin; ROSUVA, rosuvastatin; PRAVA, pravastatin; LOVA, lovastatin; NA, not available.

† Doubling the dosage of run-in statin.

‡ % of patients whose LDL-C <70 mg/dl.

### 1.2 The efficacy of ezetimibe plus statins on ASVD

#### 1.2.1 Ezetimibe plus statins in patients with CHD

The Improved Reduction of Outcomes: Vytorin Efficacy International Trial (IMPROVE-IT, NCT00202878) was the first trial to demonstrate that the addition of ezetimibe to standard statin therapy incrementally reduced the risk of CV events [44]. The trial compared the effects of simvastatin 40 mg plus ezetimibe 10 mg with simvastatin 40 mg plus placebo in 18,144 patients who had been hospitalized for an acute coronary syndrome (ACS) within the preceding 10 days and had LDL-C levels of 50 to 100 mg/dl if they were receiving lipid-lowering therapy or 50 to 125 mg/dl if they were not receiving lipid-lowering therapy. The trial showed that adding ezetimibe to statin therapy further reduced LDL-C levels. More importantly, the incidence of primary endpoint, a composite of cardiovascular death, re-hospitalization for unstable angina, nonfatal myocardial infarction, coronary revascularization, and nonfatal stroke, was significantly lower in the combination therapy (32.7% vs. 34.7%; P=0.016). The risk reduction was in accord with the predicted effects of statins, even in the low LDL-C levels, and no adverse events were observed.

The PRECISE-IVUS (Plaque Regression with Cholesterol Absorption Inhibitor or Synthesis Inhibitor Evaluated by Intravascular Ultrasound; NCT01043380) trial compared the effects of ezetimibe 10 mg plus atorvastatin and atorvastatin alone on the lipid profile and coronary atherosclerosis in Japanese patients with a history of percutaneous coronary intervention (PCI). Atorvastatin was up titrated till LDL-C <70 mg/dl. The trial employed serial volumetric intravascular ultrasound (IVUS) to quantitatively reflect the changes in coronary plaque. Compared with atorvastatin alone, ezetimibe plus atorvastatin resulted in more drastically LDL-C reduction (63.2±16.3 mg/dl vs. 73.3±20.3 mg/dl; P<0.001), a greater absolute change in percent atheroma volume (PAV) (-1.4% vs. -0.3%; P=0.001), and a higher percentage of patients with coronary plaque regression (78% vs. 58%; P=0.004) [45]. Whereby, compared to statin monotherapy, ezetimibe plus statin can further decrease LDL-C and attenuate atherosclerosis.

#### 1.2.2 Ezetimibe plus statins in patients with aneurysm of the abdominal aorta

Aneurysm of the abdominal aorta (AAA) is considered as a particular, specifically localized form of atherothrombosis, providing an accessible model of human atherothrombotic progression towards clinical events [46]. Studies suggested that statins might reduce aneurysm growth rates. Modulation of abdominal aortic aneurysm (AAA) expansion by statins might be related to reducing interleukin (IL)-6 and matrix metalloproteinase (MMP)-9, which might be consequent on the reduction of plasma cholesterol. Whereby, Dawson et al. conducted a pilot study comparing the biological effects of ezetimibe plus simvastatin to simvastatin alone on parameters relevant to aneurysm expansion including inflammatory cytokines and proteolytic enzymes [47]. Eighteen patients with large AAA scheduled for elective open aneurysm surgery were randomized to receive simvastatin 40 mg plus ezetimibe 10 mg or simvastatin 40 mg plus placebo for 32.5 days until the day of surgery. Total concentrations of tumour necrosis factor-α (TNF-α), IL-1β, -6, -8, -10, MMP-1, -2, -3, -8, -9, -12, -13, tissue inhibitors of matrix metalloproteinase (TIMP)-1 and -2 were measured in plasma, aortic wall homogenates and tissue culture explants. In this study, ezetimibe combined with simvastatin significantly reduced MMP-9 and IL-6 concentrations in the aortic wall compared with simvastatin alone, which was associated with a reduction in plasma total cholesterol and non-HDL concentrations. However, concentrations of other inflammatory cytokines or proteolytic enzymes were similar in both groups. In addition, due to the small sample size and short duration of treatment, the results of the trial are inconclusive and insufficient to clarify the efficacy of ezetimibe in combination with statins in the treatment of patients with AAA. Much further research focusing on aneurysm growth rates or AAA rupture risk is needed.

#### 1.2.3 Ezetimibe plus statins in patients with CVD

For CVD, ezetimibe plus statins could also get benefit. A parallel-group trial enrolled a total of 2,860 patients and followed for a median of 3.5 years to compare two LDL-C targets using intensive lipid-lowering therapy after transient ischemic attack (TIA) and ischemic stroke of atherosclerotic origin, all the patients had evidence of cerebrovascular or coronary-artery atherosclerosis and received a statin, ezetimibe, or both [48]. In this study, they randomly assigned patients with ischemic stroke in the previous 3 months or a TIA within the previous 15 days to a target LDL-C level of less than 70 mg/dl (lower-target group) or to a target range of 90 mg to 110 mg/dl (higher-target group). The composite primary end point of major cardiovascular events included ischemic stroke, myocardial infarction, and new symptoms leading to urgent coronary or carotid revascularization, or death from cardiovascular causes. The results revealed that after an ischemic stroke or TIA with evidence of atherosclerosis, patients who had a target LDL-C level of less than 70 mg/dl had a lower risk of subsequent cardiovascular events than those who had a target range of 90 mg to 110 mg/dl. Moreover, the incidence of intracranial hemorrhage and newly diagnosed diabetes did not differ significantly between the two groups. Another study induced by Bohula et al. investigated the efficacy of the addition of ezetimibe to simvastatin for the prevention of stroke and other adverse cardiovascular events in IMPROVE-IT, with a focus on patients with a stroke before randomization. The results revealed that addition of ezetimibe to simvastatin in patients stabilized after acute coronary syndrome reduces the frequency of ischemic stroke, with a particularly large effect seen in patients with a prior stroke [49].

#### 1.2.4 Ezetimibe plus statins in patients with PAD

A study revealed the effect of ezetimibe on peripheral arterial atherosclerosis depends on statin use at baseline. The results showed that statin initiation with or without ezetimibe in statin-naïve patients halts the progression of peripheral atherosclerosis. When ezetimibe is added to patients previously on statins, peripheral atherosclerosis progressed. Therefore, the effect of ezetimibe on peripheral atherosclerosis may depend on relative timing of statin therapy [50]. Moreover, another study showed that no significant differences were observed between mono-therapy using simvastatin and triple-therapy with simvastatin, extended-release niacin, and ezetimibe for 24-month changes in peripheral artery wall, lumen, and total vessel volumes [51]. Whereby, further study is still needed on this aspect with large number of sample size.

#### 1.2.5 Ezetimibe plus statins in patients with aortic stenosis

Aortic stenosis (AS) is a common disorder in the elderly, and it is associated with a remarkable increase in the morbidity and mortality of cardiovascular events. A large number of studies have indicated that the progression of aortic stenosis may resemble atherosclerosis. The Simvastatin and Ezetimibe in Aortic Stenosis (SEAS) trial (NCT00092677) evaluated the efficacy of long-term intensive lipid-lowering therapy with simvastatin 40 mg and ezetimibe 10 mg in 1,873 patients with mild-to-moderate asymptomatic aortic stenosis with a mean age of 67 years. The primary outcome was a composite of major cardiovascular events, including death from cardiovascular causes, aortic-valve replacement, nonfatal myocardial infarction, hospitalization for unstable angina pectoris, heart failure, coronary artery bypass grafting (GABG), PCI, and non-hemorrhagic stroke. Secondary outcomes were events associated with aortic-valve stenosis and ischemic cardiovascular events. During a median follow-up of 52.2 months, the mean percentage of LDL-C lowering was 53.8% in the simvastatin plus ezetimibe group, compared with 3.8% in the placebo group (P<0.001). Despite the great LDL-C reduction, the combination therapy had no effect on primary outcome or on aortic-valve events, while significantly reduced the risk of ischemic cardiovascular events (P=0.02), mainly due to a significant decline in the need for CABG procedures. In addition, there was no difference between the two groups in the annualized changes in the mean (±SE) peak aortic-jet velocity, which was a key echocardiographic measure to evaluate the progression of aortic stenosis, with 0.15±0.01 m per second per year in the simvastatin plus ezetimibe group and 0.16±0.01 m per second per year in the placebo group [52]. However, A post hoc analysis suggested that the effect of simvastatin combined with ezetimibe on delaying AS progression increased with higher pretreatment LDL levels and lower baseline peak aortic jet velocity [53].

#### 1.2.6 Ezetimibe plus statins in patients with CKD

Patients with chronic kidney disease (CKD) are at high risk for arterial disease. The second United Kingdom Heart and Renal Protection (UK-HARP-II) Study enrolled 263 patients (152 predialysis patients with creatinine levels no less than 1.7 mg/dl [150 mmol/L], 18 patients on peritoneal dialysis therapy, and 33 patients on hemodialysis therapy) and randomly assigned them to receive simvastatin 20 mg plus ezetimibe 10 mg or simvastatin 20 mg plus placebo. The 6-month study showed that the addition of ezetimibe to simvastatin was well tolerated and produced an incremental 21% reduction in LDL-C levels (P<0.0001), with no significant effects on triglycerides or HDL-C levels. Moreover, the addition of ezetimibe was not associated with an increased risk of abnormal liver function test results or of elevated creatine kinase levels. In conclusion, the study demonstrated that the addition of ezetimibe to simvastatin as initial therapy for CKD patients was safe and effective in treating dyslipidemia [54]. The Study of Heart and Renal Protection (SHARP; NCT00125593) trial evaluated the efficacy and safety of simvastatin 20 mg plus ezetimibe 10 mg in more than 9,000 patients with advanced CKD (serum creatinine levels ≥1.7 mg/dl in men or ≥1.5 mg/dl in women) and without a history of myocardial infarction or coronary revascularization. Compared with placebo, simvastatin plus ezetimibe significantly decreased LDL-C levels by an average of 32 mg/dl during a mean follow-up of 4.9 years. Most importantly, the SHARP trial showed that simvastatin plus ezetimibe produced a 17% reduction in major atherosclerotic events, a composite of non-fatal myocardial infarction, coronary death, non-hemorrhagic stroke, or any arterial revascularization procedure, in patients with advanced CKD [55]. Thus, patients with CKD may benefit from adding ezetimibe to statin therapy.

#### 1.2.7 Ezetimibe plus statins in patients with diabetes

According to NCEP ATP III Guidelines which were released in May 2001, diabetes were included in CHD risk equivalents which carry a risk for a major coronary event considered to be equal to that of patients with established CHD [2]. The Stop Atherosclerosis in Native Diabetics Study (SANDS) firstly compared the effects of aggressive and standard treatment of lipids and blood pressure (BP) in 499 American Indians over 40 years old with type 2 diabetes mellitus (T2DM) and with no history of CV events [56]. In the 3-year trial, participants were randomized to receive standard treatment with a goal of LDL-C ≤100 mg/dl, non-HDL-C ≤130 mg/dl, and BP ≤130/85 mmHg, or aggressive treatment with a goal of LDL-C ≤70 mg/dl, non-HDL-C ≤100 mg/dl, and BP ≤115/75 mmHg. Ezetimibe was added to subjects who failed to achieve their LDL-C goals with statins alone, while fish oil, fenofibrate, or niacin was added to manage non-HDL-C levels. Subjects in the aggressive treatment group received an average of 1.42 lipid-lowering drugs and roughly a third received ezetimibe as an adjunct to statin therapy. The aggressive treatment resulted in significant decline in carotid-artery intima-media thickness (CIMT; -0.017 vs. 0.041 mm, P<0.0001), arterial mass (-0.14 vs. 1.14 mm^2^, P<0.0001), and left ventricular mass index (LVMI; -2.4 vs. -1.3 g/m^2.7^, P=0.05), compared with the standard treatment. The secondary analyses of this trial further suggested that CIMT regression appeared to be much more related to LDL-C reduction (P<0.0005), whereas the decrease in LVMI appeared to be more closely associated with systolic BP reduction (P=0.002) [57]. The finding of SANDS suggested that lower goals of LDL-C and BP can be attained successfully and safely and, more importantly, supported the utility of ezetimibe in clinical practice.

## 2. Safety profile of ezetimibe plus statin therapy

Considerable evidence has shown that ezetimibe plus statin therapy was well tolerated, with a safety profile similar to statin monotherapy [16, 41, 44, 58, 59].

### 2.1 Muscle toxicity

A common and major cause of statin intolerance and discontinuation is statin-related myopathy, which ranges from myalgia (muscle ache or weakness without creatine kinase [CK] elevation) to myositis (muscle symptoms with CK elevation usually <10 times the upper limit of normal [ULN]) and to rhabdomyolysis (muscle symptoms with significant CK elevation generally >10 times ULN and with creatinine elevation with brown urine and urinary myoglobin) [10]. Several large-scale trials demonstrated that compared with placebo or statin alone, ezetimibe combined with a statin did not increase the incidence of muscle-related adverse events [16, 44, 52, 59]. Furthermore, a systematic review of 18 randomized controlled trials involving 14,471 participants showed that compared with statin monotherapy, combination therapy with ezetimibe and a statin did not significantly increase the risks of myalgia, CK elevation, and rhabdomyolysis [60]. Since the fact that ezetimibe plus low-dose statins could produce a comparable or larger reduction in LDL-C to high-dose statins and that statin-related myopathy is dose-dependent [36, 37, 39], ezetimibe plus low-dose statins appears to be a potential strategy to not only markedly reduce LDL-C level and CV risk, but also decrease statin-related adverse effects. More importantly, in the 2016 Guidelines for the Management of Dyslipidemias of European Society of Cardiology (ESC) and European Atherosclerosis Society (EAS), ezetimibe plus a low-dose statin or second- or third-line statin was suggested to cope with statin-related muscle symptoms [61].

### 2.2 Liver toxicity

Elevated aspartate transaminase (AST) or alanine transaminase (ALT) was a common reason for statin discontinuation previously. However, statin-related elevations in hepatic aminotransferase levels do not indicate liver injury or dysfunction. According to Hy’s law, drug-induced liver injury must meet following criteria: elevation in AST or ALT ≥3 times ULN, increase in total bilirubin levels >2 times ULN, and no other demonstrable cause such as cholestasis, viral hepatitis, pre-existing or acute hepatobiliary disease, or another drug which can cause the same injury. Additionally, the US Food and Drug Administration (FDA) has drawn a conclusion that serious liver injury related to statins is rare and unpredictable in individual patients, and that routine periodic monitoring of liver enzymes does not seem to be effective in detecting or preventing serious liver injury. Liver enzyme tests are merely recommended being performed prior to statin use and after clinical indications [62]. Many clinical trials showed that ezetimibe plus statins did not yield consecutive elevation in AST or ALT ≥3 times ULN [16, 36, 37, 39, 44, 54, 58]. In addition, a systematic overview of eighteen randomized controlled trials showed that combination ezetimibe and statin therapy did not significantly increase the risk of elevated transaminase, as compared with statins alone [60]. Moreover, there is no evidence that the addition of ezetimibe to statins significantly increase the incidences of hepatitis, jaundice, and other liver dysfunction in clinical setting.

### 2.3 Renal toxicity

Recently, Bae et al. assessed the effect of ezetimibe-statin combination therapy on renal outcome compared with statin monotherapy by analyzing longitudinal data of 4,537 patients treated with simvastatin 20 mg plus ezetimibe 10 mg (S + E) or simvastatin 20 mg alone (S) for over 180 days. Changes in serum creatinine and incidence of renal events, defined as doubling of serum creatinine to ≥1.5 mg/dl or occurrence of end-stage renal disease, were compared between the groups. Among 3,104 propensity-score-matched patients with a median follow-up of 4.2 years, the risk of renal events in the S + E group was significantly lower than that in the S group (hazard ratio 0.58; 95% CI 0.35-0.95, P= 0.032). Additionally, as assessed by changes in serum creatinine, the S + E group tended to preserve renal function compared with the S group throughout follow-up (P-values for time-group interactions<0.001). These results support the beneficial effects of ezetimibe plus statins on renal function [63]. However, further study with larger sample size is still in needed.

### 2.4 New-onset *diabetes*

Clinical trials, which evaluated the relationship between statin use and new-onset diabetes showed conflicting results. In 2001, the West of Scotland Coronary Prevention Study (WOSCOPS) reported that pravastatin 40 mg/d led to a 30% risk reduction in new-onset diabetes but used non-standardized criteria for diabetes diagnosis [64]. In contrast, in 2008, the Justifification for the Use of statins in Prevention: an Intervention Trial Evaluating Rosuvastatin (JUPITER) trial showed that a 25% increasing in the relative risk of newly diagnosed T2DM was observed in rosuvastatin 20mg/d [65]. Subsequent meta-analyses of data from randomized statin trials showed that statin therapy was associated with a modest but significant increase in risk for incident T2DM, with higher risks for more intensive statin therapy [66-69]. For example, in 2010, a meta-analysis of 13 large, randomized statin trials with 91,140 participants indicated that statin therapy increased the incidence of new-onset diabetes by 9% [69]. Furthermore, in 2012, FDA indicated that statin therapy increases the levels of glycated hemoglobin A1c (HbA1c) and fasting blood glucose (FBG). However, the benefit of statins in reducing the risk of CV events far outweighs the disadvantage of statins in increasing the incidence of new-onset diabetes, with several fewer major CV events occurring for each excess case of new-onset diabetes associated with intensive or non-intensive statin therapy [70]. These results highlight the importance of evaluating the effect of adding ezetimibe to statin therapy on glycemic control. The effect of ezetimibe in combination with statins on glucose metabolism is uncertain [24, 40, 71-74]. Dagli et al. found that low-dose pravastatin plus ezetimibe (10/10 mg) is superior to high-dose pravastatin (40 mg) in improving insulin resistance in patients with primary hyperlipidemia and known CHD or 10 years CHD risk > 20% [71]. Moutzouri et al. showed that simvastatin 40 mg, rosuvastatin 10 mg and simvastatin 10 mg plus ezetimibe 10 mg were correlated with insulin resistance, and there was no significant difference between groups [73]. Kishimoto et al. concluded that ezetimibe combined with statins had no significant effect on the values of glucose metabolism parameters in hypercholesterolemic patients with and without diabetes [74]. A meta-analysis of sixteen randomized controlled trials indicated that ezetimibe did not adversely affect HbA1c or FBG in patients with or without diabetes. Moreover, it also showed that compared to high-dose statins, ezetimibe combined with low-dose statins for over 3 months significantly reduced FBG, suggesting that ezetimibe may partly offset the glycemic adverse effects related to statins [75].

### 2.5 Cancer

The Cholesterol Treatment Trialists’ (CTT) meta-analyses provide robust evidence that long-term statin therapy does not increase the incidence of cancer overall or at any particular site and death from cancer, even at low LDL-C levels [3, 4]. However, in the SEAS trial, there was a significant increase in cancer incidence and cancer death in the simvastatin plus ezetimibe group, compared with the placebo group, and the risk of incident cancer was not related to the extent of LDL-C reduction [52]. Thus, the results of the SEAS trial once aroused great concern that ezetimibe might increase the risk of cancer. Subsequently, two large-scale and long-term randomized controlled trials, the SHARP trial involving 9,270 participants with a median follow-up of 4.9 years and the IMPROVE-IT trial involving 1,8144 participants with a median follow-up of 6 years, showed that ezetimibe plus simvastatin did not increase the rate of cancer, as compared with placebo or simvastatin monotherapy, respectively [44, 55, 76].

### 2.6 Cognitive impairment

Studies showed a potential association between statin use and cognitive impairment in some individuals, however, the 2014 Statin Safety Task Force concluded that there was a lack of consistent quality evidence of the effect of statin therapy on cognition, whether adverse or beneficial [77]. Williams and colleagues conducted Mendelian randomization analyses using HMGCR, PCSK9, NPC1L1, and APOB gene variants that encode the protein targets of statins, proprotein convertase subtilisin/kexin type 9 (PCSK9) inhibitors, ezetimibe, and mipomersen, respectively. Then they performed a meta-analysis of Mendelian randomization estimates for LDL-C-weighted regional variants on Alzheimer’s disease (AD) risk from 2 large samples and found that the HMGCR and NPC1L1 models did not indicate that the use of related lipid-lowering drug classes would affect the risk for AD [78]. There is no evidence that adding ezetimibe to statin therapy influences cognition.

### 2.7 Hemorrhagic stroke

The Stroke Prevention by Aggressive Reduction in Cholesterol Levels (SPARCL) trial examined high-intensity statin therapy (atorvastatin 80mg/d) versus placebo in 4,731 patients with recent stroke or TIA and with LDL-C levels of 100 to 190 mg/dl and without known CHD. Although the risk of nonfatal and fatal stroke was markedly reduced, there was a slight but statistically significant increase in the incidence of hemorrhagic stroke in the atorvastatin group, compared with the placebo group [79]. The results of the SPARCL trial underscored the increased risk of hemorrhagic stroke associated with statin therapy. However, the CTT meta-analyses of randomized statin trials demonstrated that statin therapy significantly reduced the overall risk of stroke and the risk of ischemic stroke, with non-significant increase in the risk of hemorrhagic stroke [3, 4]. Furthermore, in the IMPROVE-IT trial, there was a small but not significant increase in hemorrhagic stroke (59 vs. 43; HR 1.38, 95% CI 0.93-2.04; P=0.01) in simvastatin plus ezetimibe group, as compared with simvastatin group [44]. Likewise, in the SHARP trial, non-significantly more patients with haemorrhagic stroke were observed in simvastatin 20 mg plus ezetimibe 10 mg group, compared with placebo group (45 [1.0%] vs. 37 [0.8%]; RR 1.21, 95% CI 0.78-1.86; P=0.4) [55]. However, potentially additional risk of hemorrhagic stroke is far outweighed by the substantial reductions in the risk of overall stroke and major cardiovascular events.

**Table 3:** Mean percent changes in lipid parameters and biomarkers from baseline to week 12 in phase III bempedoic acid plus statins trials.

Trials	Mean baseline LDL-C (mg/dl)	Percent change (%) from baseline to week 12 (bempedoic acid vs. placebo; between groups difference [95% CI]; P-value)						
		LDL-C	Non-HDL-C	TC	Apo-B	Hs-CRP	TG	HDL-C
CLEAR Harmony NCT02666664 2019 [81]	103.2	-16.5 vs. 1.6	-11.9 vs. 1.5	-10.3 vs. 0.8	-8.6 vs. 3.3	-22.4 vs. 2.6 ^*^	2.9 vs. -0.3 ^*^	-5.9 vs. 0.1
		-18.1 (-20.0, -16.1)	-13.3 (-15.1, -11.6)	-11.1 (-12.5, -9.8)	-11.9 (-13.6, -10.2)	-21.5 (-27.0, -16.0)	NA	NA
		P<0.001	P<0.001	P<0.001	P<0.001	P<0.001	NA	NA
CLEAR Serenity NCT02988115 2019 [82]	157.6	-23.6 vs. -1.3	-19.0 vs. -0.4	-16.1 vs. -0.6	-15.5 vs. -0.2	-25.4 vs. 2.7 ^*^	7.9 vs. 7.4	-5.2 vs. -0.6
		-21.4 (-25.1, -17.7)	-17.9 (-21.1, -14.8)	-14.8 (-17.3,-12.2)	-15.0 (-18.1 to -11.9)	-24.3 (-35.9, -12.7) ^†^	0.4 (-8.2, 9.0)	-4.5 (-7.5, -1.6)
		P<0.001	P<0.001	P<0.001	P<0.001	P<0.001	P=0.921	P=0.003
CLEAR Wisdom NCT02991118 2019 [83]	120.4	-15.1 vs. 2.4	-10.8 vs. 2.3	-9.9 vs. 1.3	-9.3 vs. 3.7	-18.7 vs. -9.4 ^*^	11.0 vs. 6.1	-6.4 vs. -0.2
		-17.4 (-21.0, -13.9)	-13.0 (-16.3, -9.8)	-11.2 (-13.6, -8.8)	-13.0 (-16.1, -9.9)	-8.7 (-17.2, -0.4) ^†^	4.9 (-1.5, 11.3)	-6.1 (-8.4, -3.9)
		P<0.001	P<0.001	P<0.001	P<0.001	P=0.04	P=0.13	P<0.001
CLEAR Tranquility ^‡^ NCT03001076 2018 [84]	127.6	-23.5 vs. 5.0	-18.4 vs. 5.2	-15.1 vs. 2.9	-14.6 vs. 4.7	-32.5 vs. 2.1 ^*^	-1.4 vs. 7.8 ^*^	-7.4 vs. -1.4
		-28.5 (-34.4, -22.5)	-23.6 (-26.4, -20.8)	-18.0 (-20.0, -16.0)	-19.3 (-21.6, -17.0)	-31.0 ^†^	NA	NA
		P<0.001	P<0.001	P<0.001	P<0.001	P<0.001	NA	P=0.002

Data are least-squares mean ± standard errors, unless otherwise specified. LDL-C, low-density lipoprotein cholesterol; Non-HDL-C, non-high-density lipoprotein cholesterol; TC, total cholesterol; Apo-B, apolipoprotein B; hs-CRP, high-sensitivity C-reactive protein; HDL-C, high-density lipoprotein cholesterol.

* Data are medians.

† Data are location shifts (asymptotic confifidence limits).

‡ Patients in this trial were randomized to treatment with bempedoic acid 180 mg or placebo once daily added to ezetimibe 10 mg/day. Patients in other trials were randomized to treatment with bempedoic acid 180 mg or placebo once daily.

## 3. Safety and efficacy of ezetimibe plus statins and bempedoic acid

Bempedoic acid (8-hydroxy-2,2,14,14-tetramethyl-pentadecanedioic acid; ETC-1002) is a novel agent on reducing LDL-C. ETC-1002 is a pro-drug converted to an active metabolite (ETC-1002-CoA) by liver acyl-CoA synthetase, which inhibits ATP citrate lyase (ACL), an enzyme located upstream of HMG-CoA reductase (the target of statins) in the mammalian cholesterol biosynthesis pathway. By inhibiting cholesterol synthesis in the liver, bempedoic acid induces the up-regulation of LDL receptor and stimulates the uptake of LDL by the liver, thereby reducing LDL-C levels [80]. The effects of bempedoic acid on lipid parameters and biomarkers were revealed in several phase III clinical trials (Table 3) [81-84].

The CLEAR Tranquility (NCT03001076) trial firstly demonstrated the efficacy and safety of the addition of bempedoic acid to statins and ezetimibe in patients with statin intolerance who require additional LDL-C lowering. After a 4-week ezetimibe 10 mg/d run-in period, patients were randomized to receive bempedoic acid 180 mg (n=181) or placebo (n=88) once daily added to ezetimibe 10 mg/d for 12 weeks. Significant reductions in LDL-C, non-high-density lipoprotein cholesterol (Non-HDL-C), total cholesterol (TC), apolipoprotein B (Apo-B), and high-sensitivity C-reactive protein (hs-CRP) were observed in bempedoic acid group versus placebo group (all P<0.001). Furthermore, the addition of bempedoic acid was well tolerated, with rates of treatment-emergent adverse events, muscle-related adverse events, and discontinuations similar to the placebo group [84]. Moreover, another phase III trial (NCT03337308) showed that the fixed-dose combination of bempedoic acid 180 mg and ezetimibe 10 mg was more effective in improving lipid profile than bempedoic acid 180 mg alone, ezetimibe 10 mg alone or placebo in hypercholesterolemic patients at high risk for cardiovascular disease treated with maximally tolerated statin therapy, with a similar safety profile [85]. Whereby, bempedoic acid provided a therapeutic option complementary to ezetimibe in patients who are statin intolerant or receiving maximally tolerated statin therapy and require additional LDL-C lowering.

### Discussion

From all studies mentioned above, we conclude that ezetimibe plus statins may benefit not only for CHD, but also for all ASVD subtypes and related diseases. Further research is still needed to explore the exact mechanism and the costumed therapeutic strategy of this combination therapy.

Statins and ezetimibe work in different mechanisms, the combination of ezetimibe and statins is synergistic. Adding ezetimibe to low-dose statins can achieve the equal or higher lipid-lowering effect of high-dose statins alone with fewer adverse events; especially suitable for those who cannot achieve their LDL-C goals even underwent statins with intensive dose or maximum tolerable dose.

Ezetimibe plus statins may diminish statin-related adverse effects. For elder patients who require PCI or CABG but have contraindications (such as severe hepatic and renal dysfunction), ezetimibe plus statins may be the more proper therapeutic option.

Ezetimibe plus statins is a cost-effective and well-tolerated strategy to reduce the risk of ASVD events. Therefore, patients who are able to attain their LDL-C targets with high-dose statins alone may also use ezetimibe plus low-dose statins.

For patients who require additional LDL-C lowering, especially for those who are statin intolerant or receiving maximally tolerated statin therapy, ezetimibe plus statins and/or bempedoic acid therapy may be a proper therapeutic option. Muti-drug combination therapies may be future directions for these patients.

### Conclusion

Ezetimibe plus statins may contribute to preventing ASVD safely and effectively. However, whether it should be applicable to all populations still needs further research.

### Limitations

This article has some limitations: Firstly, it only outlines the clinical studies about the efficacy and safety of adding ezetimibe to statin therapy. Secondly, the cases number included in the studies enrolled in this artcile was not large enough. Last but not least, the detailed mechanisms of ezetimibe plus statins are not described in this article.
